# A bibliometrics analysis and visualization study of TRPV1 channel

**DOI:** 10.3389/fphar.2023.1076921

**Published:** 2023-03-21

**Authors:** Ning Gao, Meng Li, Weiming Wang, Zhen Liu, Yufeng Guo

**Affiliations:** ^1^ Department of Acupuncture and Moxibustion, Guang’anmen Hospital, China Academy of Chinese Medical Sciences, Beijing, China; ^2^ Department of Gastroenterology, Guang’anmen Hospital, China Academy of Chinese Medical Sciences, Beijing, China; ^3^ Department of Gastroenterology, Xiyuan Hospital, China Academy of Chinese Medical Sciences, Beijing, China

**Keywords:** TRPV1, bibliometric, research hotspots, VOSviewer, citespace

## Abstract

**Background:** At the end of the 1990s, transient receptor potential vanilloid 1 (TRPV1) was first identified and cloned, serving as a key pain and heat sensor in humans. A large body of evidence have revealed its polymodal structure, complex function and wide-spread distribution, the specific mechanism of the ion channel remains unclear. Our goal here is to perform a bibliometric analysis and visualization study to present hotspots and trends in TRPV1 channel.

**Materials and Methods:** TRPV1-related publications from inception to 2022 were retrieved from the Web of Science database. Excel, VOSviewer, and CiteSpace software were utilized for co-authorship, co-citation and co-occurrence analysis.

**Results:** There were 9,113 publications included in the study, the number of publications increased rapidly after 1989, from 7 in 1990 to 373 in 2007, during which the number of citations per publication (CPP) also reached a peak in 2000 (CPP = 106.52). A total of 1,486 journals published TRPV1 articles, mainly belong to Q1 or Q2 divisions; The United States published the most articles (TP = 3,080), followed by Japan (TP = 1,221), China (TP = 1,217), and England (TP = 734); In recent years, the TRPV1-related research direction has been broaden to multiple fields related to inflammation, oxidative stress, and apoptosis; Keyword clustering refined the topic distributions and could be generalized as neuralgia, endogenous cannabinoid system, TRPV1 mediated airway hyperresponsiveness, involvement of apoptosis, TRPV1 antagonists as therapy targets.

**Conclusion:** By conducting an exhaustive bibliographic search, this review refined the topic distributions and generalized as neuralgia, endogenous cannabinoid system, TRPV1 mediated airway hyperresponsiveness, involvement of apoptosis, TRPV1 antagonists as therapy targets. It is currently being clarified how exactly TRPV1 works as an ion channel, and much more in-depth basic research is needed in the future.

## Introduction

At the end of the 1990s, transient receptor potential vanilloid 1 (TRPV1) was first identifed and cloned (Caterina et al., 1997), a non-selective ion channel belong to the transient receptor potential (TRP) channel superfamily, and served as a key pain and heat sensor in humans (Caterina et al., 2000) TRPV1 is widely expressed by primary afferent nerve fibres of the dorsal root ganglion (DRG), trigeminal ganglion (TG) neurons (Iftinca et al., 2021), and vagal nodose ganglia, in which it functions as a key nociceptive channel in signal transduction in multiple processes. It could be activated by a wide spectrum of physical and chemical stimuli including noxious heat (>43°C) (Kwon et al., 2021), divalent cations (Cao et al., 2014) such as Mg^2+^ and Ba^2+^, low pH, inflammatory mediators (Iftinca et al., 2021), as well as animal toxins (Geron et al., 2017). Sensitivity and response to multiple stimuli enables TRPV1 to function as a polymodal protein in multiple cell types, tissues, and organs (Tominaga et al., 1998a; Benítez-Angeles et al., 2020).

The last years of the TRPV1 structural analysis research have seen growth, albeit interaction sites with the protein have been decoded merely for some of these molecules (Benítez-Angeles et al., 2020). TRPV1 as a non-selective tetrameric cation channel (Niemeyer, 2005), the three-dimensional structure is similar to voltage-gated ion channels (Liao et al., 2013), with each subunit consisting of 838 amino acids, of which residues 433–684 form the transmembrane region (Moiseenkova-Bell et al., 2008a). The receptor has different sizes of cytoplasmic N and C termini (Clapham, 2003). The transmembrane region includes S1-S6 with six helices, S1-S4 forming the voltage sensor-like structural domain and S5-S6 forming the inner pore region (Bevan et al., 2014). Advances in cryoelectron microscopy have made it possible to obtain structures sufficiently precise to resolve edge chain conformations (Carnevale and Rohacs, 2016). The three-dimensional structure of TRPV1 was clarified by cryoEM, with a height of 150 Å, consisting of a small region accounting for 30% of the total volume and a larger basket-shaped region, probably corresponding to the transmembrane region and the N- and C-terminal portions of the cytoplasm, respectively (Moiseenkova-Bell et al., 2008b). TRPV1 is activated by many endogenous and exogenous compounds including animal toxins (Yang et al., 2015), acidic environment (Tominaga et al., 1998b), and Mg^2+^ (Yang et al., 2014). In the activated state, the pore-forming spirals are separated from each other, the hydrophobic seal is significantly damaged, and the lower gate is opened (Cao et al., 2013).

A large body of evidence have revealed the presence and activity of this ion channel in several cell types or tissues, like numerous cancer cell types (Li et al., 2021), cardiac afferent fibers (Hori et al., 2021), and spermatozoa, etc., (Ramal-Sanchez et al., 2021). Studies indicates a pivotal role of TRPV1 in pain and pruritus development (Fernández-Carvajal et al., 2022). Additionally, TRPV1 activation also impacts on mitochondrial functions (Juárez-Contreras et al., 2020), mediates Anandamide’s regulation of sperm function (Xiao and Chen, 2022), and serve as relevant target for metabolic interventions (Panchal et al., 2018). In summary, due to its polymodal structure, complex function and wide-spread distribution, the specific mechanism of the ion channel as well as the elucidation of its roles are currently being clarified.

Bibliometrics is a thorough method that combines quantitative and qualitative analyses to reveal a variety of features of publications, including identifying the countries, journals, authors, and institutions that contribute to a research area, displaying frequently cited studies and keywords, and establishing the collaboration between those countries, institutions, and authors in a particular scientific research field (Diane Cooper, 2015). To date, bibliometric analysis has been widely used in medical sciences, such as dermatology (Zhang et al., 2022a), oncology (Xiao et al., 2022), and neurology (Zhang et al., 2022b).

To the best of our knowledge, there has been no bibliometric analysis on the topic of TRPV1 channel published until now. To fill this gap, this bibliometric analysis conducted a global map of the scientific publications on TRPV1 channel related research from the inception to 2022 using CiteSpace, VOSviewer, and Excel, in order to summarize and illustrate the temporal features, spatial features, content and state of this ion channel over the years. It may also help researchers to quickly identify the research hotspots and cutting-edge trends in the TRPV1 field in recent years. This study will therefore focus on the following 3 research questions:

(1) What is the basic distribution of the TRPV1 field, such as annual publication volume, authors, countries, institutions, etc.?
(2) What are the hot directions in the field of TRPV1?
(3) What are the research trends in the field of TRPV1?

## Methods

### Data sources and search strategies

In this study, the WoS core collection database, which has the longest coverage time and high-quality database, was selected as the data source. All publications related to TRPV1 were obtained from the Web of Science Core Collection (WOSCC). The search strategy was as follows: TS = (TRPV1) OR TS = (vanilloid receptor 1) OR TS = (VR1) OR TS = (capsaicin receptor). In addition, there is no restriction on the publication date of the publication. Studies were excluded if they were non-English and non-article publications. Three authors were independently searched and screened. If in doubt, disagreements were resolved *via* discussion or arbitration by a fourth reviewer if necessary.

### Data collection

The data retrieved and extracted from WoSCC database, including title, author, country, journal, keywords, etc., were downloaded in “.txt” and “.xls” formats, respectively, and then imported into VOSviewer (version.1.6.18; The Center for Science and Technology Studies. Netherlands), Microsoft Excel (version. 2019; Microsoft Corporation; Washington, United States), and Citespace (version.V; Drexel University; Pennsylvania, United States). The data imported into citespace were saved in the format of “download_***”. To avoid bias caused by day-to-day database updates, all literature searches and data exports were performed on the same day (06 October 2022).

### Statistical tools

A total of 10 statistical tools were utilized in this study, including 7 bibliometric indicators, such as Price’s law, Bradford’s law, Participation Index(PaI); 3 Bibliometric software, respectively Microsoft Excel, Citespace, VOSviewer. Source, formula and usage of these 10 statistical tools are presented in Table1.

**TABLE 1:** Bibliometric tools used in the research.

Bibliometric tools	Source or formula	Use
Price’s law	$y\left(x\right)=ae^{bx}$	Common indicators reflecting the laws of scientific production Price (1963)
Bradford’s law	$Core:Zone1:Zone2:Zone3=1:a:a^{2}:a^{3}$	—
Impact factor (IF)	https://www.medsci.cn/sci/index.do	A vital indicator of a journal’s impact Ellegaard and Wallin (2015)
Participation Index(PaI)	$PaI=\frac{Number\;of\;publications\;in\;a\;country}{Total\;number\;of\;publications}\times 100$	—
Citations per publication(CPP)	$CPP=\frac{Total\;publications\;\left(TP\right)}{Total\;citations\;\left(TC\right)}$	—
Microsoft Excel 2019	Microsoft Corporation	Descriptive statistics for publications Yu et al. (2020)
Citespace V	Drexel University	Collating the history of the growth of the research topic Chen (2006)
VOSviewer 1.6.18	The Center for Science and Technology Studies	Web application for building knowledge maps Liu et al. (2022)

### Statistical analysis

Price’s law was used to analyze the annual distribution of publications and to assess whether they were in the exponential growth phase by coefficient of determination (R2). Bradford’s law was adopted to identify the most prolific core journals in the TRPV1 field. Quantitative analysis of data such as frequency, percentage and ranking of authors, journals, countries/regions, etc., was performed using Microsoft Excel. Excel was also used to create maps depicting the distribution of publications around the world and statistical charts illustrating the number of publications per year. VOSviewer is used to build a collaborative network of authors, countries/regions, and institutions in order to study collaborative linkages and the intensity of collaboration of different nodes, as well as to perform clustering analysis of frequently co-cited references and keywords. CiteSpace is employed in the creation of keyword timelines and bursts. The following parameters were set: time span (inception-2022), Year Per Slice (1), Node Types (Keyword), g-index (k = 25), and pruning (Pathfinder).

## Results

**FIGURE 1:**
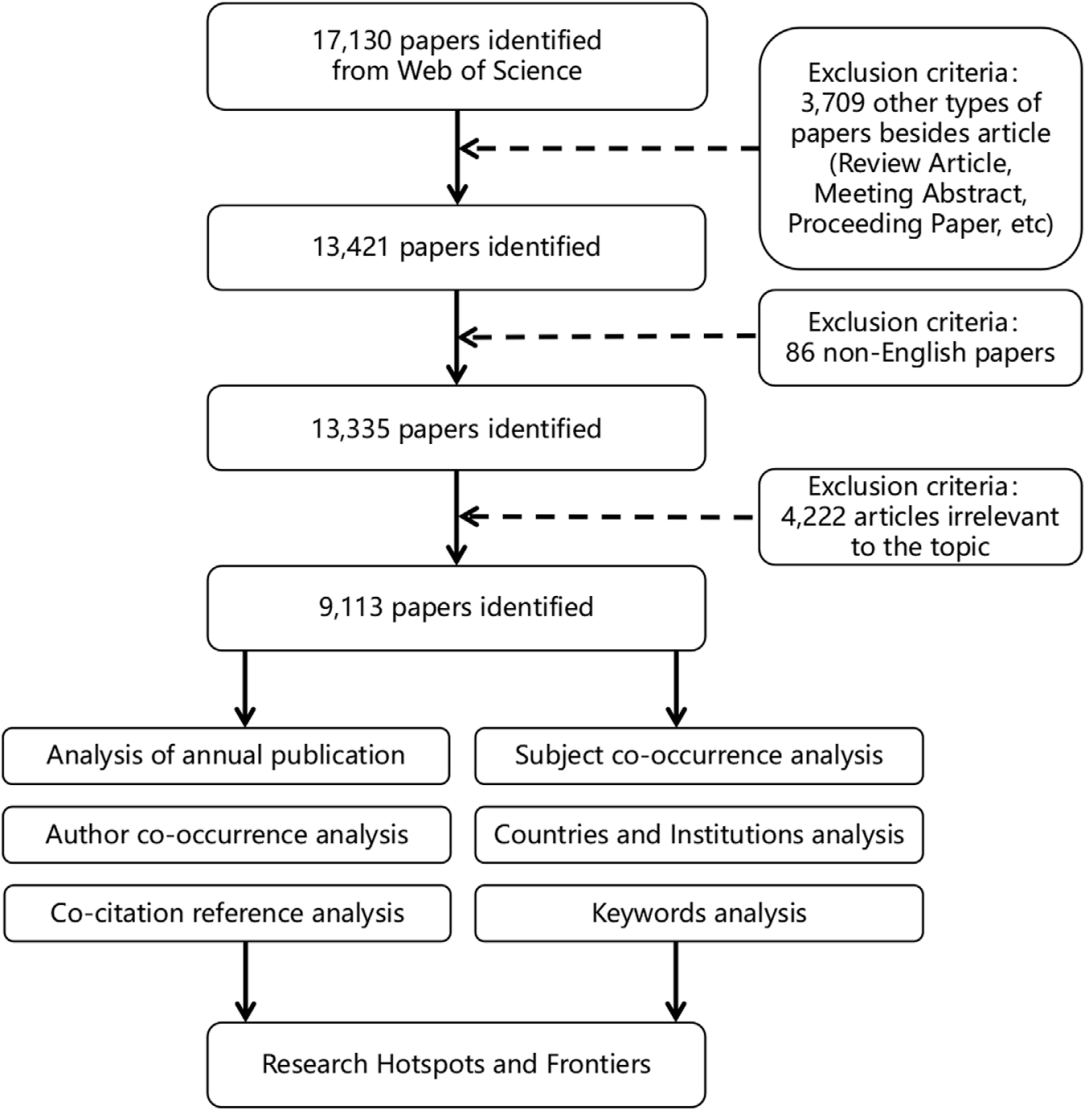
Flow chart of literature screening.

A total of 17,130 studies were identified through an initial search. After removing non-English and non-article publications, 13,335 studies were reviewed by title and abstract, and 4,222 studies were excluded for irrelevant to the topic. The details of the study selection process were shown in Figure 1.

### Publication outputs and citation trend

**FIGURE 2:**
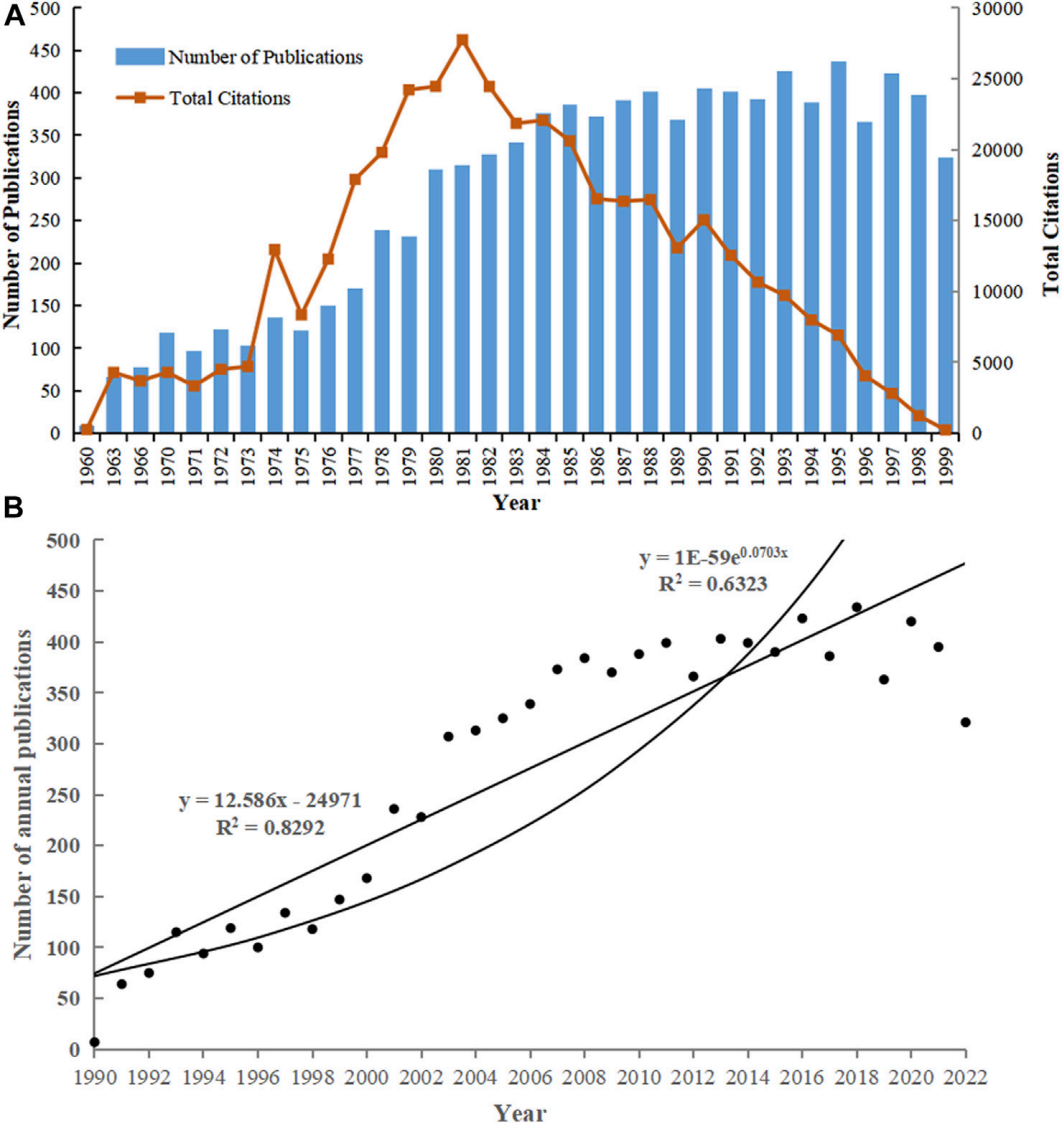
Analysis of the number and trends of publications. **(A)** Annual number of the publications and citations in TRPV1 research. **(B)** Fitted curves for annual number of publications.

The increasing volume of literature in a field over the years generally indicates the continued interest of scholars in that field of study. Citation counts usually mean the value of content published, and also represent the degree of attention and recognition by researchers in the related field. There were 9,113 publications included in the study, however, before 1989 research was in a nascent stage, the number of publications is relatively limited and lack continuity. The number of publications increased rapidly after 1989, from 7 in 1990 to 373 in 2007, with an average annual increase of 21.53, in the exploratory phase of research, during which CPP also continued to grow and reached a peak in 2000 (CPP = 106.52). The period from 2008 to 2022 is in the maturity stage of the study, and the annual number of articles fluctuates in the range of 363–434 and tends to be stable, with an average of 389.40 articles per year, except for 2022, when the counting has not yet ended (Figure 2A). Studies have shown that TRPV1 has been intensively studied over the past over 50 years and is still a popular area of research. It can be observed that the number of citations to articles gradually decreases, as the number of articles published each year increases, indicating we also need to improve the quality of research, as we conduct extensive research.

To identify whether the growth in research output followed Price’s law, we exponentially adjusted the data using the equation *y* = 1E-59e^0.0703x^ with 36.77% of the variance not explained by the model fit (*R* ^2^ = 0.6323). We adjusted the data linearly again using the equation *y* = 12.586x - 24971, which had a variance of 17.08% (*R* ^2^ = 0.8292) (Figure 2B). Compared to the exponential fit, our data can be better fit to the linear fit.

In 1969, researchers Cosens and Manning observed a sophophora melanogaster mutant and found that the mutant showed a transient increase in intracellular calcium ion concentration by continuous light stimulation (Cosen and Manning, 1969). In 1989, Craig Montell of the University of California, Berkeley, discovered that this was due to a mutation in an ion channel-like membrane protein in sophophora melanogaster, so they cloned the gene first and named the protein “TRP channel” (Montell and Rubin, 1989). TRPV1-related studies gradually increased after 1989.

### Most cited documents

Citation numbers are used to measure an article’s impact over time by counting the number of times it has been mentioned (Hirsch, 2005). We list the top 10 most cited articles (Table 2), of which the most cited article was published by MJ. Caterina, David Julius, etc., in 1997 (Caterina et al., 1997). It was the first time that TRPV1 was identifed and cloned, which demonstrates clearly that proteinacious ion channels are the molecular target of capsaicin activation on sensory neurons. It was also found that thermal stimuli within the noxious temperature range likewise activate the cloned capsaicin receptor. The second most cited article was also published in 2000 by MJ. Caterina, David Julius, et al. (Caterina et al., 2000). By this time, the major factors that can sensitize VR1 channels, such as vanilloid compounds, protons, or heat (>43°C), had been largely identified, and furthermore, the essential role of VR1 in mediating selective pain perception and tissue injury-induced thermal nociceptive hypersensitivity has been shown to be necessary (Caterina et al., 2000). The third most cited paper was published in 1998 by M Tominaga, David Julius, et al. (Tominaga et al., 1998a). The study found that protons can lower the temperature excitation threshold of VR1, i.e., moderately acidic conditions (pH ≤ 5.9) can activate VR1 at room temperature, and VR1 is regarded as a molecular integrator of chemical and physical stimuli that cause pain (Tominaga et al., 1998a). The fourth most cited literature was published in 2002 by DD McKemy, David Julius, and et al. (McKemy et al., 2002). The investigators identified and cloned a menthol receptor, CMR1, from trigeminal sensory neurons that is sensitive to cold and menthol and is thought to function as a transducer of cold stimuli in the somatosensory system (McKemy et al., 2002). These findings, together with the previous identification of heat-sensitive channels such as VR1, demonstrate that TRP channels can detect temperature over a wide range and are major receptors of thermal stimuli in the mammalian peripheral nervous system. The fifth high-cited article was published in 2003 by GM Story, Ardem Patapoutian, et al. (Story et al., 2003). The discovery of a cold-activated channel co-expressed with VR1, ANKTM1, which is activated at a lower temperature than CMR1 and can also be activated by capsaicin, further broadens the physiological spectrum of temperature perception in mammals (Story et al., 2003).

**TABLE 2:** The top 10 high-cited papers in TRPV1 research.

Rank	First author	Journal	Title	TC
1	MJ Caterina	Nature	The capsaicin receptor: a heat-activated ion channel in the pain pathway	6787
2	MJ Caterina	Science	Impaired nociception and pain sensation in mice lacking the capsaicin receptor	2784
3	M Tominaga	Neuron	The cloned capsaicin receptor integrates multiple pain-producing stimuli	2478
4	DD McKemy	Nature	Identification of a cold receptor reveals a general role for TRP channels in thermosensation	1897
5	GM Story	Cell	ANKTM1, a TRP-like channel expressed in nociceptive neurons, is activated by cold temperatures	1885
6	PM Zygmunt	Nature	Vanilloid receptors on sensory nerves mediate the vasodilator action of anandamide	1787
7	AM Peier	Cell	A TRP channel that senses cold stimuli and menthol	1612
8	SE Jordt	Nature	Mustard oils and cannabinoids excite sensory nerve fibres through the TRP channel ANKTM1	1490
9	JB Davis	Nature	Vanilloid receptor-1 is essential for inflammatory thermal hyperalgesia	1424
10	M Bandell	Neuron	Noxious cold ion channel TRPA1 is activated by pungent compounds and bradykinin	1391

### Contributions of countries and institutions

**FIGURE 3:**
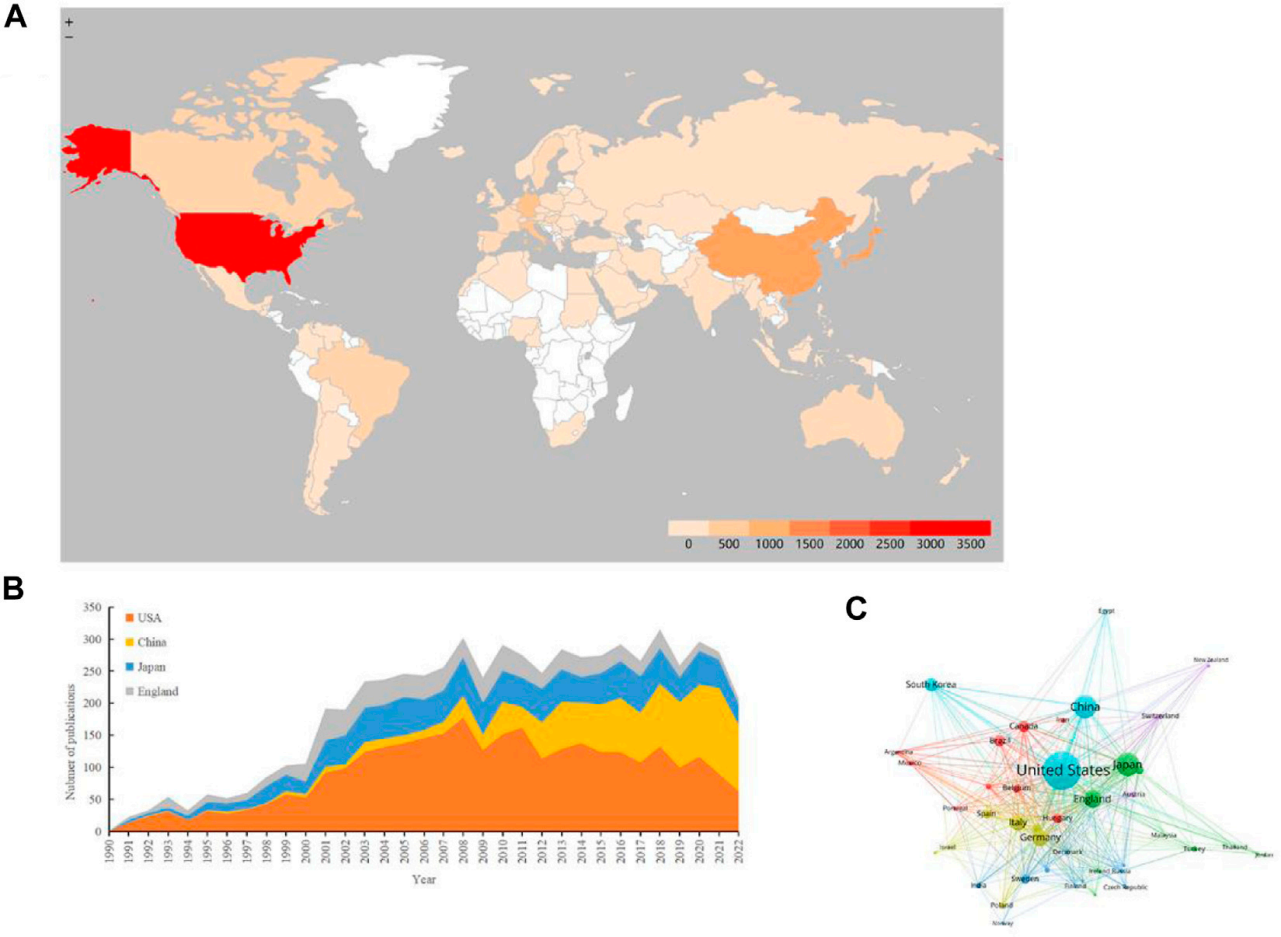
The distribution of countries or regions in TRPV1 research. **(A)** Distribution of TRPV1 publications in the world map. According to the color gradient in the lower right corner, the color of each country or region represents the amount of literature published. **(B)** The distribution trend of the top four countries or regions by year. **(C)** Map of cooperation network between countries or regions. Different color blocks represent different countries or regions, the area of each color block represents the volume of literature published in the country or region, and the thickness of the connection between the color blocks indicates the frequency of cooperation between countries or regions.

To better understand the academic contributions of different countries to the TRPV1 field, this section analyzed the contribution of individual countries in the global TRPV1 study. Between 1968 and 2022, 91 countries contributed to TRPV1 publications, and the distribution is shown in Figure 3A, and the top 10 countries in terms of contribution are summarized in Table 3, with the United States publishing the most articles (PaI = 33.80), followed by Japan (PaI = 13.40), China (PaI = 13.35), and England (PaI = 8.05). The annual number of publications in these countries is shown in Figure 3B, with the United States, Japan, and England growing rapidly from 1990, reaching peaks in 2008, 2008, and 2001, respectively. The research in this field in China began relatively late, and yet it has developed rapidly. The analysis of international cooperation indicates that there is extremely close cooperation among countries around the world, with the United States being the country most frequently involved in global cooperation (Figure 3C).

**TABLE 3:** The top 10 countries contributing to publications in TRPV1 research.

Rank	Country	TP	PaI	TC	CCP
1	United States	3080	33.80	185553	60.24
2	Japan	1221	13.40	40026	32.78
3	China	1217	13.35	22891	18.81
4	United Kingdom	734	8.05	50164	68.34
5	Germany	651	7.14	30939	47.53
6	Italy	596	6.54	32399	54.36
7	Republic of Korea	439	4.82	14073	32.06
8	Brazil	342	3.75	7854	22.96
9	Canada	339	3.72	12315	36.33
10	Hungary	244	2.68	7379	30.24

**FIGURE 4:**
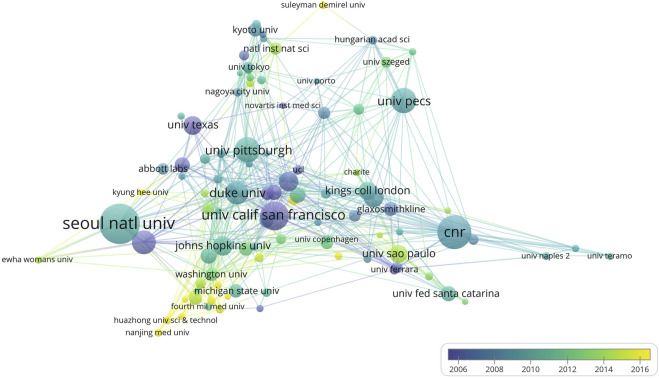
Co-authorship overlay visualization map of institutions. The color of each circle corresponds to the average year of publication, the size of the circle is proportional to the number of publications, and the thickness of the connecting lines between the circles indicates the frequency of collaboration between institutions.

The top 10 institutions in terms of the number of publications by TRPV1 are shown in Table 4, with Seoul National University having the highest number of publications (TP = 199), followed by National Research Council (TP = 164), and University of California San Francisco (TP = 138). Six of the top 10 producing institutions are situated in the United States, which indicates that the United States has been a target country for research on TRPV1. The institutional cooperation network was constructed with the threshold value set to 30 (Figure 4), which showed the cooperation relationship among 102 institutions, and it can be seen that there is closer cooperation between institutions in the field of TRPV1, which contributes to the academic communication and cooperation.

**TABLE 4:** The top 10 most productive institutions in TRPV1 research.

Rank	Institution	Country	TP	Percentage (%)
1	Seoul National University	Republic of Korea	199	2.18
2	National Research Council	Italy	164	1.80
3	University of California San Francisco	United States	138	1.51
4	University of Pittsburgh	United States	123	1.35
5	University of Pecs	Hungary	121	1.33
6	National Cancer Institute	United States	113	1.24
7	Duke University	United States	111	1.22
8	University of Erlangen-Nuremberg	Germany	96	1.05
9	Johns Hopkins University	United States	93	1.02
10	Harvard University	United States	91	1.00

### Contributions of authors

The author who published the most papers was Dr. Vincenzo Di Marzo, a research director from endocannabinoid research group of institute of biomolecular chemistry in Italy. Dr. Vincenzo Di Marzo mainly focuses on the effects of endogenous cannabinoid system (Cristino et al., 2020), especially the biological basis of cannabinoids in the field of neurology, mechanism of action, pharmacological properties and its conduction pathways, demonstrates that cannabinoids can stimulate TRPV1 and other signaling pathways to mediate anti-inflammatory, immunomodulatory and neuroprotective effects (Keimpema et al., 2021). The second prolific author was Peter M Blumberg from the Laboratory of Cancer Biology and Genetics, National Cancer Institute Cancer Research Center, United States, shared a close collaboration with Jeewoo Lee of the Laboratory of Medicinal Chemistry, Institute of Pharmaceutical Sciences, College of Pharmacy, Seoul National University. They are mainly engaged in the research of targeted analgesia (Lee et al., 2019) and structural analysis (Thorat et al., 2021) of TRPV1 agonists or antagonists. Makoto Tominaga, who is a professor in the Department of Cellular and Molecular Pharmacology at the University of California, San Francisco, is from the same institution as David Julius, the 2021 Nobel Laureate in Physiology or Medicine, and is the author with the highest total number of citations. In 1997, David Julius innovatively applied expression cloning to find a capsaicin-activated protein molecule, VR1, and discovered that VR1 can be activated by heat (Caterina et al., 1997). From the discovery of the receptor to further studies of its structural and functional relationships, the gradual finding of the receptor’s analgesic effects as a clinical intervention target has contributed greatly to the advancement and evolution of the field. Pierangelo Geppetti, M.D., Professor of Clinical Pharmacology at the University of Florence and Director of the Headache Center at Careggi University Hospital, focuses on the major contributing pathways in migraine mechanisms (Benemei et al., 2014). It is proposed by Pierangelo Geppetti that TRPA1 is activated by pain-inducing exogenous and endogenous drugs that release pro-migraine peptides and calcitonin gene-related peptides through this neuronal pathway, which mediated the onset of headache (Benemei et al., 2014). It was also found that the calcitonin gene-related peptide (CGRP)-mediated neuronal/Schwann cell pathway facilitated allodynia associated with neurogenic inflammation, which contributes to the analgesic effect of CGRP in mice (De Logu et al., 2022).

**FIGURE 5:**
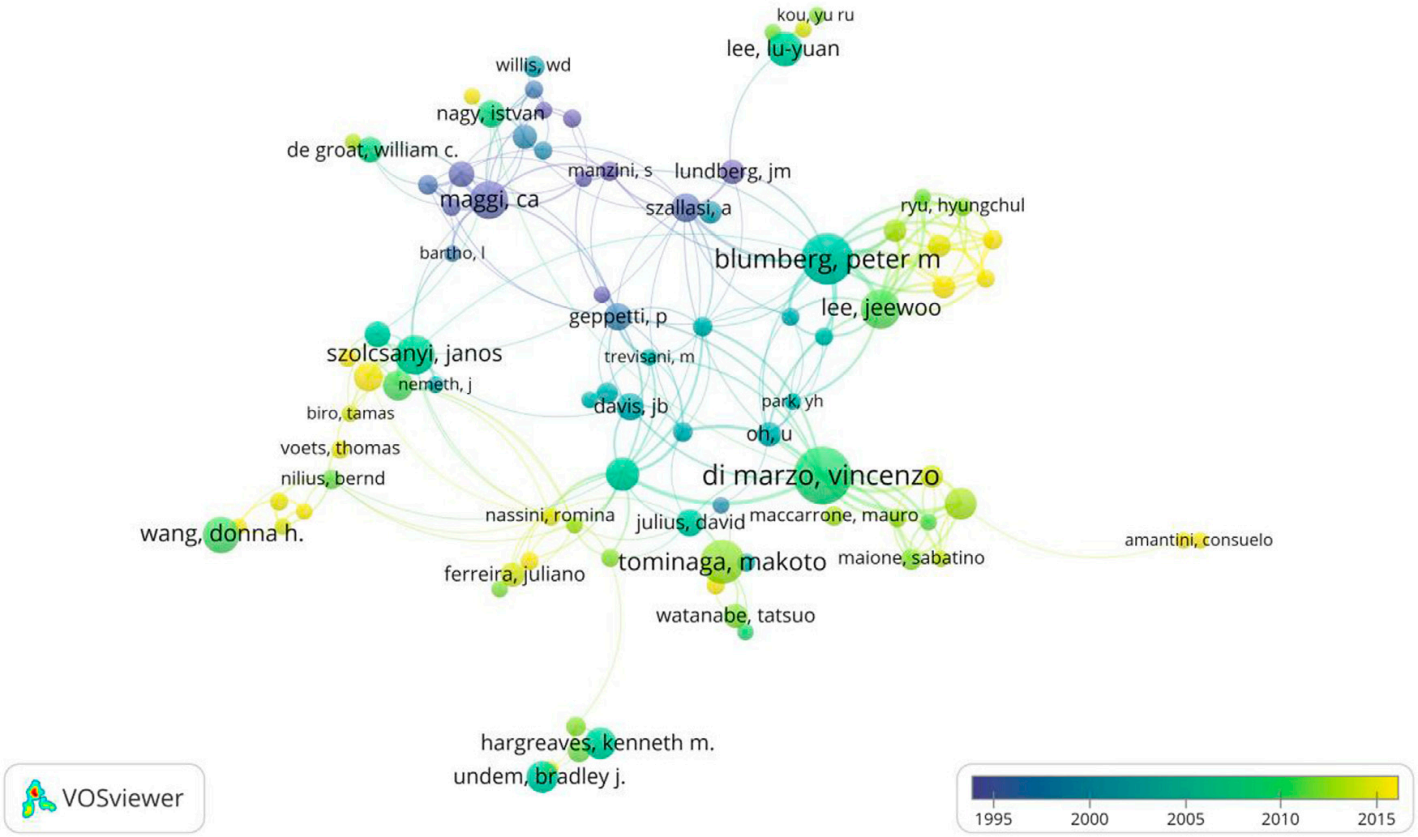
Co-authorship overlay visualization map of authors. The color of each circle corresponds to the average year of publication of the author, the size of the circle is proportional to the number of papers published by the author, and the thickness of the connecting line indicates the frequency of collaboration.

The number of researchers’ publications reflects the academic level and research ability of the individual researcher to a certain extent. Table 5 presents the 10 most productive authors in the field of TRPV1, with Vincenzo Di Marzo from Canada in first place (TP = 128), followed by Peter M Blumberg from National Cancer Institute, United States (TP = 109), Makoto Tominaga from Mie University School of Medicine, United States (TP = 81) and Jeewoo Lee from College of Pharmacy, Seoul National University, Korea (TP = 68). Makoto Tominaga (TC = 16212) and Vincenzo Di Marzo (TC = 12394) have the most total citations and are prominent in the field. In terms of author collaborations (Figure 5), authors in this field are closely linked to each other, with Carlo Alberto Maggi, S Giuliani, and A Szallasi conducting research in this field before 2000, and in recent years, many new forces of research have emerged, such as Romina Nassini, Sun Choi, and Zhiming Zhu.

**TABLE 5:** The top 10 most productive authors in TRPV1 research.

Rank	Author	TP	Percentage (%)	TC
1	Vincenzo Di Marzo	128	1.40	12394
2	Peter M Blumberg	109	1.20	4849
3	Makoto Tominaga	81	0.89	16212
4	Jeewoo Lee	68	0.75	1537
5	János Szolcsányi	66	0.72	2599
6	Carlo Alberto Maggi	63	0.69	2127
7	Donna H Wang	58	0.64	1513
8	Lu-Yuan Lee	55	0.60	2121
9	Pierangelo Geppetti	51	0.56	6987
10	L De Petrocellis	47	0.52	1978

### Journal analysis

We evaluated journals in the TRPV1 field by Bradford’s model, which shows (Table 6) that a total of 1,486 journals have published articles on TRPV1. Journals are categorized into five Bradford’s zones based on the number of articles published, with an average of 1823 articles per zone. Although the core contains only nine journals (0.61%), it publishes more than 20% of the literature. In addition, we found that Core:Zone 1:Zone 2:Zone 3 ˜ 1:3:3^2^:3^3^, except for Zone 4, which is in accordance with Bradford’s law.

**TABLE 6:** Distribution of the journals in Bradford’s zones.

	No. of journals	% Of journals	No. of articles	% Of articles	Bradford multiplier
Core	9	0.61	1891	20.75	
Zone 1	23	1.55	1778	19.51	2.56
Zone 2	71	4.78	1794	19.69	3.09
Zone 3	225	15.14	1827	20.05	3.17
Zone 4	1158	77.93	1823	20.00	5.15
Total	1486	100.00	9113	100.00	3.49

Based on Bradford’s model, we identified nine core journals in the TRPV field, including four American journals, 3 Dutch journals, and two British journals (Table 7). BRITISH JOURNAL OF PHARMACOLOGY has the largest number of articles, 308, and the highest impact factor of these 10 journals. Seven of the ten journals belong to Q1 or Q2 divisions. The above information all indicate that the overall research in the field of TRPV1 is of high quality and has received attention and recognition from high level journals.

**TABLE 7:** The top 9 core journals in TRPV1 research.

Rank	Journal	Country	TP	TC	CCP	JCR	If
1	British Journal of Pharmacology	United Kingdom	308	15818	51.36	Q1	9.473
2	European Journal of Pharmacology	Netherland	293	8588	29.31	Q2	5.20
3	Journal of Neuroscience	United States	229	27176	118.67	Q1	6.71
4	Pain	United States	215	11808	54.92	Q1	7.93
5	Neuroscience	Netherland	214	9093	42.49	Q3	3.71
6	Neuroscience Letters	Ireland	167	5165	30.93	Q3	3.20
7	Journal of Pharmacology and Experimental Therapeutics	United States	158	9776	61.87	Q2	4.40
8	Plos One	United States	155	4655	30.03	Q2	3.75
9	Brain Research	Netherland	152	4702	30.93	Q3	3.61

### Research areas analysis

The frequency analysis of the study areas reveals what topics have been driven by the research. The top 10 research areas associated with TRPV1 research according to the frequency are summarized in Table 8, with Neurosciences & Neurology, Pharmacology & Pharmacy, and Biochemistry & Molecular Biology being the three areas with the highest number of studies. The subsequent also involves anesthesiology, gastroenterology, respiratory, endocrine and metabolic aspects, showing the wide spectrum of the channel study with the complicated roles. Neurosciences & Neurology and Pharmacology & Pharmacy have no doubt to be the first two research areas, as The distribution as well as the interaction of TRPV1 can be used as a target of pharmacological intervention for pain relief. Gastroenterology & Hepatology, Respiratory System, and Endocrinology & Metabolism are three relatively new areas of research related to TRPV1, which shows that the functional applicability of the receptor is being discovered and extended to new areas of research through continuous investigation.

**TABLE 8:** The top 10 research areas in TRPV1 research.

Rank	Research area	TP	Percentage (%)
1	Neurosciences & Neurology	2772	30.42
2	Pharmacology & Pharmacy	2280	25.02
3	Biochemistry & Molecular Biology	1031	11.31
4	Physiology	1012	11.11
5	Anesthesiology	386	4.24
6	Gastroenterology & Hepatology	332	3.64
7	Cell Biology	535	5.87
8	Research & Experimental Medicine	359	3.94
9	Respiratory System	279	3.06
10	Endocrinology & Metabolism	257	2.82

### Co-occurrence analysis of keywords

**FIGURE 6:**
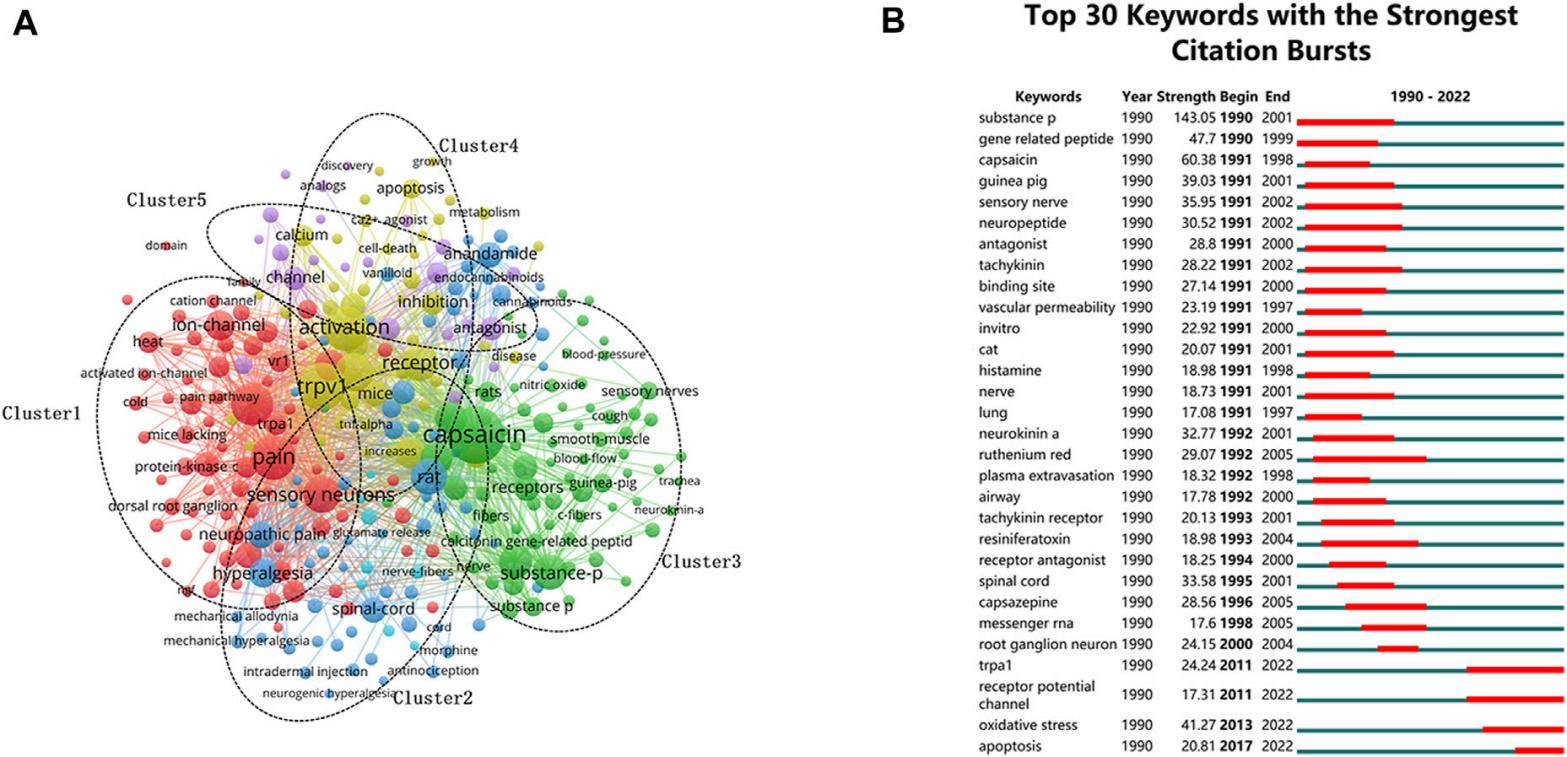
Analysis of keywords. **(A)** Map of keyword clustering in TRPV1 research. **(B)** Top 30 keywords with the strongest citation bursts.

Keyword co-occurrence networks are created by taking the keywords of an article as individual nodes, and each co-occurrence of a pair of keywords is modeled as a link between their respective nodes (Yuan et al., 2022). The co-occurrence frequency of each keyword pair is expressed as the weight of the link connecting the keyword pair (Yuan et al., 2022). The threshold of literature quantity was set to 60, and the keyword co-linear network visualization graph was generated (Figure 6A), containing 231 keywords, which were summarized as follows: 1) Cluster1 (red), neuralgia; 2) Cluster2 (blue), analgesic effects of endogenous cannabinoid system; 3) Cluster3 (green), asthma-related research; 4) Cluster4 (yellow), apoptosis; 5) Cluster5 (purple), antagonists as therapeutic targets. Major keywords ranked among the 20 top keywords, except for the search terms, include: capsaicin, sensory neurons, substance-p, gene-related peptide, inflammation, hyperalgesia, neuropathic pain, anandamide, receptor antagonist, and spinal-cord, etc., (Table 9).

**TABLE 9:** The top 20 keywords in TRPV1 research.

Rank	Keyword	TP	Rank	Keyword	TP
1	TRPV1	3596	11	Inflammation	709
2	Capsaicin	2870	12	Ion-channel	664
3	Pain	1621	13	Hyperalgesia	585
4	Expression	1141	14	Neuropathic pain	520
5	Sensory neurons	1050	15	Nociception	503
6	Rat	987	16	Anandamide	471
7	Substance-p	904	17	Receptors	456
8	Gene-related peptide	791	18	Receptor antagonist	454
9	Neurons	744	19	Spinal-cord	450
10	Mechanisms	729	20	Mice	426

CiteSpace’s burst detection feature helps to identify research topics with steep citation increases in a short period of time, which can reflect the hot disciplines of research and their shifting trends in different periods. Figure 6B presents 30 keywords with high burst citation scores in the TRPV1 research area, where the red bars indicate the time periods of citation surges. Both the intensity and duration of bursts vary, reflecting the evolving content and direction of TRPV1 research. As shown by the temporal distribution of burst keywords, in recent years, TRPV1-related functional studies have not only been limited to the perception of the sensation of pain and temperature, but also broaden to multiple fields related to inflammation, oxidative stress, and apoptosis.

## Discussion

According to the results of this study, TRPV1 research started to emerge in the medical academia since the 1960s, and the number of research publications has been increasing annually, and the academic attention has also started to grow. In general, the trend of the number of TRPV1 publications is generally in accordance with its research history: from the early 1970s to the end of 1980s, TRPV1 research was in the nascent stage, and the receptor was gradually recognized and named, since then the receptor began to receive attention, and relevant research publications began to arise, but the number of publications at this stage was few and discontinuous. After the 1990s, the number of papers in this field has been increasing.

This study illustrates the research focus and findings in different periods, the development process and trends of the field, etc. According to the publication outputs, keyword clusters, burst citation scores, and the time periods of citation surges, we enumerated the TRPV1 research hotspots and domains from the following perspectives: neuralgia, endogenous cannabinoid system, TRPV1 mediated airway hyperresponsiveness, involvement of apoptosis, TRPV1 antagonists as therapy targets.

### TRPV1 and neuralgia

TRPV1 ion channels, which are highly expressed in nociceptive DRG neurons, are well-established polymodal receptors for pain sensation (Julius, 2013). It is known that after TRPV1 activation, nociceptors release a variety of neuropeptides, including substance P and CGRP, which activate secondary neurons in the dorsal horn of the spinal cord and trigger biochemical cascades at the periphery that result in neurogenic inflammation (Iftinca et al., 2021). In the condition of inflammation, TRPV1 channels are also activated by various pro-inflammatory factors, such as prostaglandins, serotonin (5-HT), bradykinin, histamine, CGRP α, tumor necrosis factor *α* (TNFα), and etc., (Iftinca et al., 2021), which all contribute to the occurrence of neuralgia.

### Endogenous cannabinoid system

Over the last 25 years, the endocannabinoid system (ECS) has emerged as a significant neuromodulatory system (Lu and Mackie, 2016). Cannabinoid receptors, endogenous cannabinoids (endocannabinoids), and enzymes responsible for endocannabinoid synthesis and degradation comprise the ECS (Lu and Mackie, 2016). CB1 cannabinoid receptors are the most common, but cannabinoids also activate CB2 cannabinoid receptors, TRP channels, and peroxisome proliferator activated receptors (PPAR’s) (Lu and Mackie, 2016). TRPV1 channels are activated by cannabinoids, and the functional co-expression has been reported between cannabinoid receptors and TRPV1 channels (Tsumura et al., 2012). These receptors mediates several cell functions, such as bone metabolism, sensory transduction (Tsumura et al., 2012), and anti-inflammatory effects on rheumatoid arthritis (RA) (Lowin and Straub, 2015). Inhibition of TRPV1 function by concomitant CB1 activation and anandamide (AEA)-induced desensitization (atty acid amid hydrolase inhibition) might be a promising strategy to reduce RA disease activity and pain (Lowin and Straub, 2015).

### TRPV1 mediated airway hyperresponsiveness

The cough reflex is regulated by vagal, and airway sensory neurons, including a population of nociceptors expressing TRPV1 and TRPA1 channels, on which activated can evoke cough (Bonvini and Belvisi, 2017). It was observed that expression of TRPV1 channels is raised in patients with chronic persistent cough (Groneberg et al., 2004). In addition to the cough reflex, activation of TRPV1-expressing sensory nerves in the airways also elicits reflex bronchoconstriction and mucus secretion mediated through cholinergic pathways (Lee et al., 2011). Treatment directed at TRPV1 significantly alleviated airway hyperresponsiveness, airway inflammation, and remodeling in a chronic asthma murine model, which indicated the TRPV1 receptor can be a potential drug target for chronic bronchial asthma (Choi et al., 2018).

### Involvement of apoptosis

The imbalance between proliferation and apoptosis may induce cancer formation, thus anti-cancer therapies shift the balance in the opposite direction by reducing proliferation and upregulating apoptosis (Zhai et al., 2020). Mitochondrial dysfunction and membrane depolarization, endoplasmic reticulum stress, caspase activation, and DNA damage are all implicated in TRPV1-mediated apoptosis (Kahya et al., 2017). TRPV1, a ligand-activated membrane ion channel, functions in both apoptotic cell death and proliferation (Li et al., 2021), constitutes a promising target in anti-cancer therapies (Zhai et al., 2020).

### TRPV1 antagonists as therapy targets

The channel is considered to be a promising target for developing modality-specific drugs to treat pain and other TRPV1-associated disorders. Capsaicin, the agonist of TRPV1, could selectively activate TRPV1, inducing Ca^2+^ influx and related signaling pathways (Panchal et al., 2018). Repeated activation of TRPV1 receptors promotes desensitization so that the channel is insensitive to capsaicin and other harmful stimuli, which prevents calcium overload during repeated stimulation of TRPV1, and perform an analgesic and neuroprotective effect (Diane Cooper, 2015; Iftinca et al., 2021). It has been suggested that the specific molecular mechanisms may be as follows. Capsaicin and resiniferatoxin-induced DRG neurons and Trpv1-expressing cells promote endocytosis and receptor downregulation *via* lysosomal degradation. This process, which appears to be regulated by an endocytotic mechanism independent of clathrin and which can be influenced by the PKA-dependent phosphorylation of serine, requires Ca^2+^ entry (Sanz-Salvador et al., 2012). Accordingly, topical administration with capsaicin creams or patches for chronic-neuropathic-pain conditions were proposed (Iftinca et al., 2021), and approved by the European Union and the US Food and Drug Administration (FDA) in the year of 2009. New capsaicin formulations and alternative modalities of TRPV1 agonists and antagonists are being investigated as a promising therapeutic strategy to treat intractable pain (Iftinca et al., 2021).

### TRPV1 and the pruritus

The use of capsaicin for the treatment of itching due to various diseases has a long history (Breneman et al., 1992; Lotti et al., 1994). Kim (Kim et al., 2004) et al. published the first study that offered pharmacological evidence for the involvement of TRP channels in itch generated by the pruritogen histamine. TPRV1 is recognized to be associated with several chronic pruritic conditions, such as psoriasis (Nattkemper et al., 2018), atopic dermatitis (Moore et al., 2018), spontaneous pruritus caused by liver failure (Belghiti et al., 2013). Histamine is the most well-known endogenous pruritogen, and its signaling is linked to TRPV1 activation, which causes membrane depolarization and the activation of Ca^2+^-dependent intracellular cascades (Kim et al., 2004; Jian et al., 2015). Research demonstrated that histamine-induced pruritus can be prevented by inhibitory effects on peripheral nerve TRPV1 receptors (Kittaka and Tominaga, 2017; Lee et al., 2018); whereas pruritus induced by non-histaminergic pruritogens such as *α*-5HT and endothelin 1 was not affected in TRPV1 knockout (KO) mice (Imamachi et al., 2009). It was also found that histamine-induced scratching behavior was reduced but not eliminated in TRPV1KO mice, suggesting that other molecules are involved in the pruritic response, such as transient receptor potential A1 (Kittaka et al., 2017), cysteinyl leukotrienes receptor 2 (Voisin et al., 2021). In addtion, phospholipase A2 and lipoxygenase, or Gq/11-PLC3, may be involved in the relationship between the histamine receptor and TRPV1 channels (Han et al., 2006; Imamachi et al., 2009).

Recent studies have revealed that, in addition to transmitting and regulating nociception, TRPV1 activation may induce anti-tumor immune effects. This study provides preliminary evidence of the anti-inflammatory effect of low-dose systemic capsaicin treatment (Erin, 2020). TRPV1 is also known to be involved in cough, asthma, pain, inflammation, pruritus, auditory sensation, taste, apoptosis, oxidative stress, and other physiological and pathological processes in the body.

There are still some limitations in this study. 1) In terms of data integrity, only literature in English was retrieved in this study, which result in selection bias in the study; 2) The search of the database was only for WOS, without searching Google scholar and Scopus, which may also lead to some missing literature; 3) Additionally, some high-quality literature may not be analyzed due to late publication or insufficient citations.

## Conclusion

This study has identified and illustrated the publication outputs, keyword clusters, burst citation scores, and the time periods of citation surges, the research focus and findings in different periods, the development process and trends of the field. In recent years, the TRPV1-related research direction has been broaden to multiple fields related to inflammation, oxidative stress, and apoptosis. Keyword clustering refined the topic distributions and could be generalized as neuralgia, endogenous cannabinoid system, TRPV1 mediated airway hyperresponsiveness, involvement of apoptosis, TRPV1 antagonists as therapy targets. The specific functional mechanisms are still unclear, and much more in-depth basic research is needed in the future.

## Abbreviations

CGRP, calcitonin gene-related peptide; CMR1, cold and menthol receptor 1; CPP, number of citations per publication; DRG, dorsal root ganglion; ECS, endocannabinoid system; FDA, food and drug administration; KO, knockout; PaI, participation index; PPAR’s, peroxisome proliferator activated receptors; RA, rheumatoid arthritis; TC, total citations; TG, trigeminal ganglion; TNFα, tumor necrosis factor *α*; TP, total publications; TRP, transient receptor potential; TRPV1, transient receptor potential vanilloid 1; USA, United States of America; VR1, vanilloid receptor 1; WOSCC, web of science core collection.
